# The levels of asymmetric dimethylarginine in patients with isolated coronary artery ectasia

**DOI:** 10.4103/0256-4947.65263

**Published:** 2010

**Authors:** Ismail Erden

**Affiliations:** Düzce University, Cardiology Department, Konuralp, Düzce, Turkey E-mail: iserdemus@yahoo.com

**To the Editor:** Coronary artery ectasia (CAE) has been defined as localized or diffuse dilation of the coronary arteries exceeding the 1.5 fold of normal adjacent segment in coronary angiography.^1^ Although coronary artery disease (CAD) is supposed to be responsible for more than 50% of coronary ectasia, the precise pathology of CAE is not clearly understood. Elevated serum levels of ADMA have been demonstrated to be associated with cardiovascular disease and many of the risk factors related with endothelial dysfunction such as hypercholesterolemia, hypertension, smoking and diabetes. Therefore, elevated serum concentrations of ADMA have been considered as an indicator of endothelial dysfunction and a risk factor for cardiovascular disease.^2^

In this study, we tested the hypothesis that endothelial dysfunction may be present in patients with CAE. Therefore, we investigated serum ADMA levels in patients with and without CAE. Forty-one consecutive patients with angiographically proven normal coronary arteries and CAE (28 men, 13 women, mean (SD) age: 54.4 (10.5) years) and forty-eight sex- and age-matched control participants with angiographically proven normal coronary arteries but without associated CAE (27 men, 21 women, mean (SD) age: 51.1 (14.1) years) were included in the study. Patients with coronary artery disease including obstructive lesions, unstable angina, any form of cardiomyopathies and any history of systemic disease were excluded from the study. No significant difference was present between the two groups regarding the use of medicine.

The baseline demographic and clinical characteristics of the patients with CAE and normal coronary flow did not differ. Serum ADMA concentrations in patients with CAE were found to be significantly higher (1.9 [0.9] μmol/l vs. 1.1 [0.7] μmol/l, *P*=.01) than those of control participants. In order to understand whether ADMA level is an independent determinant for CAE, logistic regression analysis was performed. The covariates considered were age, sex, hypertension, diabetes mellitus, hyperlipidemia, family history and cigarette smoking. The analysis showed that ADMA level is an independent determinant for CAE. [odds ratio=1.486, 95% confidence interval: 0.978-2.054; *P*=.03]. In the subgroup analyses, ADMA was higher in patients with widespread involvement with ectasia compared with mild involvement (2.1 [0.6] vs. 1.6 [0.7] μmol/L, *P*=.04).

Over the last decade, evidence has accumulated from clinical and experimental studies for a close association of elevated serum concentrations of ADMA and vascular endothelial dysfunction.^3^ The major findings of this study that the patients with CAE have higher serum concentrations of ADMA further strengthen the concept that vascular endothelial function is impaired in patients with CAE. Although this study was not designed to investigate the mechanism by which ADMA contributes to CAE, it may be concluded that, by impairing coronary flow, elevated serum concentrations of ADMA may be responsible for the myocardial ischemic symptoms and the positive results of stress test for myocardial ischemia in patients with CAE. Thus, it may be suggested that ADMA reducing therapies such as angiotensin converting-enzyme inhibitors and receptor antagonists or rosiglitazone may be helpful in the treatment of patients by improving endothelial dysfunction.^4^ To provide symptomatic relief and improve the objective findings of myocardial ischemia, much interest should be focused on the exact mechanisms and the therapeutic approaches of CAE.
